# Masquelet–Ilizarov technique for the management of bone loss post debridement of infected tibial nonunion

**DOI:** 10.1007/s00264-022-05494-y

**Published:** 2022-06-30

**Authors:** Abdullah Khaled, Osama El-Gebaly, Mahmoud El-Rosasy

**Affiliations:** Department of Orthopedic Surgery, Faculty of Medicine, Tanta University, Tanta, Egypt

**Keywords:** Ilizarov, Bone transport, Masquelet technique, Infected tibia, Nonunion

## Abstract

**Purpose:**

Masquelet and Ilizarov techniques have their advantages and shortcomings in the reconstruction of bone defects. The aim of this study was to evaluate the effectiveness of the combination of both techniques for the management of infected tibial nonunion to combine the advantages of both techniques with avoidance of shortcomings of both of them.

**Patients and methods:**

A prospective single-centre study was performed during the period from 2012 to 2019. Patients with the infected nonunion of the tibia with bone defect were included. Patients with pathological fractures or non-infected bone loss were excluded. Management protocol for all patients consisted of two stages. The first stage was Masquelet induced membrane technique and the second stage was Ilizarov bone transport. The results were assessed based on both objective (clinical and radiographic evaluation) and subjective criteria (limb function and patient satisfaction).

**Results:**

Thirty-two patients were included in this study. The mean size of the defect was 6 cm. Ilizarov bone transport was done through the induced membrane chamber in all cases with an average follow-up of 28 months. Successful reconstruction without recurrence of infection was achieved in 30 cases (94%). No other bone or soft tissue procedure was needed with satisfactory functional outcome in 27 out of 30 cases (90%). Three cases had unsatisfactory results due to leg length discrepancy, joint stiffness, and persistent pain.

**Conclusions:**

Masquelet–Ilizarov technique can be used for the management of infected nonunion tibia with high satisfactory results without the need for complex soft tissue procedures.

## Introduction 

Reconstruction of critical-sized bone defect in the infected nonunion of the tibia is very challenging and may require multiple complicated surgery. Associated soft tissue defect is common in these cases either from the injury or during the debridement due to the subcutaneous position of the tibia [1]. Several reconstructive approaches have been described in the literature including induced membrane technique (Masquelet technique), bone transport using external skeletal fixation techniques, vascularized tissue transfers (Orthoplastic techniques), and combinations of the previous techniques [2–7]. The presence of deep infection can ruin any reconstructive attempt despite an apparently adequate debridement.

The Masquelet technique has been accepted as a management of bone defects with a success of up to 25 cm defect-size in a two-stage procedure using polymethyl methacrylate (PMMA) cement in the first stage. PMMA is used as a spacer, local antibiotic delivery, and for stimulation of osteogenic membrane formation allowing for eradication of infection, improving soft tissue condition, and preparation for grafting in the second stage [8, 9]. The shortcoming of this procedure is that it does not deal with the soft tissue problem which sometimes requires complex surgery, well-equipped theatre rooms, and highly trained orthopaedic and plastic surgeons. Moreover, there are reports of recurrence of infection in cases of infected tibial nonunion treated by induced membrane technique [10, 11].

Bone transport using distraction histogenesis (Ilizarov techniques) has a wide consensus as a management of composite bone and soft tissue loss [12, 13]. Cases of infected nonunion of the tibia are frequently associated with bone osteoporosis, bad soft tissue condition, and tissue oedema. Such difficulties render using Ilizarov techniques in a single-stage procedure challenging and may yield unsatisfactory results [14].

We hypothesized that using both techniques in succession (Masquelet–Ilizarov technique) in the management of infected nonunion of the tibia can combine the advantages of each technique with avoidance of the shortcoming of both of them.

## Patients and methods

All patients with infected nonunion of the tibia during the period from 2012 to 2019 with bone (± soft tissue) defect either from the trauma or during the debridement were included in this prospective study. Patients with pathological fractures and with non-infected bone loss due to other causes were excluded. Ethical clearance was obtained from our local ethical committee. Written informed consent was obtained from all the participants. Our integrated institutional protocol for the management of infected nonunion of the tibia was applied to all included patients. This protocol consists of two stages. The first stage (Masquelet induced membrane technique) includes debridement of the infected nonunion and obliteration of the resultant dead space using antibiotic-impregnated cement spacer. In the second stage, bone and soft tissue reconstruction was performed by bone transport using Ilizarov external fixator through the induced membrane chamber.

### Surgical technique

#### Stage 1. Debridement and management of the dead space

The patient was positioned in the supine position with the surgical incision following the previous incisions direct from the skin to the bone with the avoidance of elevating a subcutaneous flap. Debridement of all necrotic bone and soft tissues with removal of the retained implant was performed. Transverse osteotomy of the bone ends was performed (square osteotomy).

**Fig. 1:**
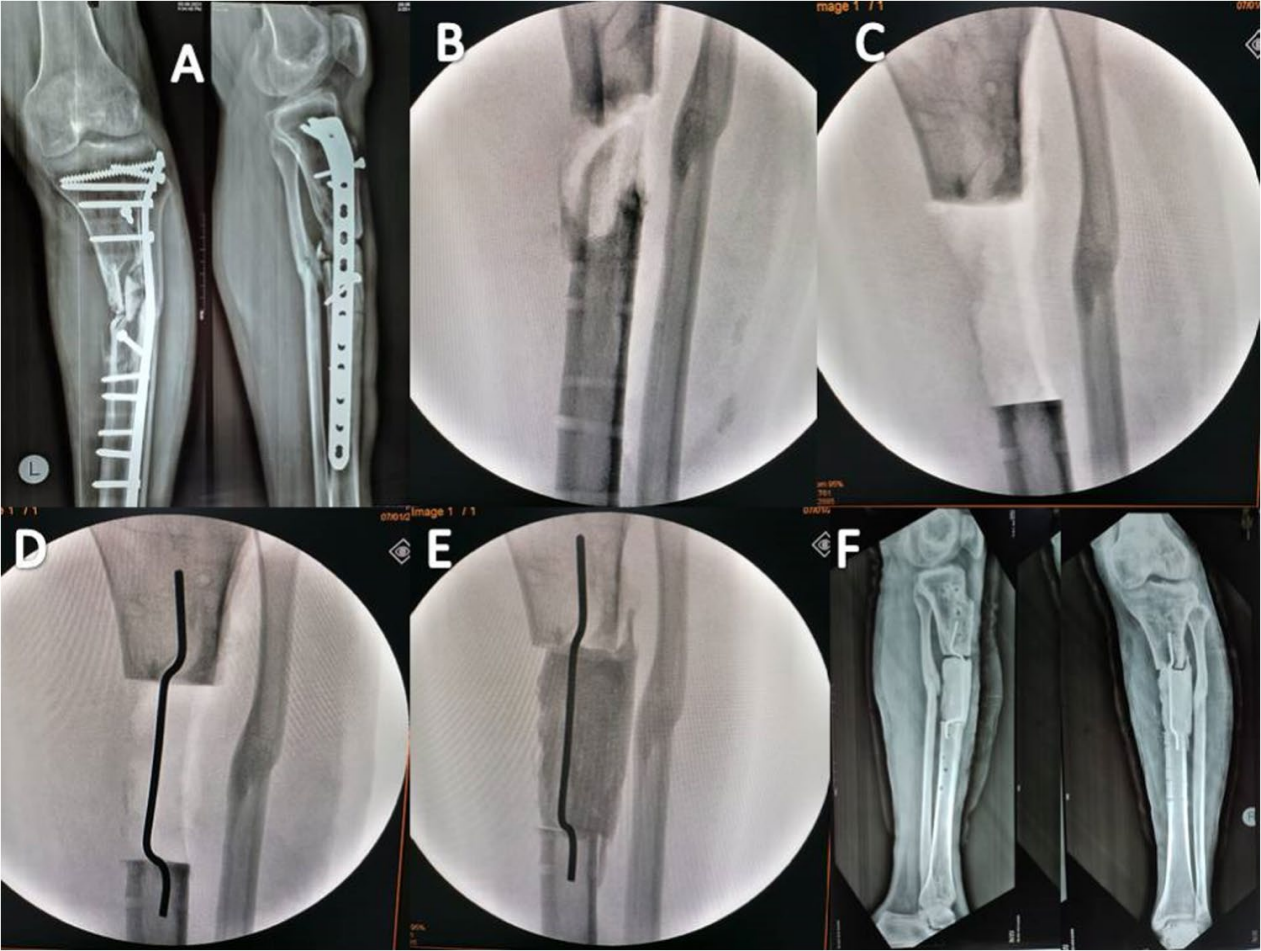
Phase 1 of surgical debridement and stage 2 of management of the dead space. **A** Ap and lateral X-ray of infected non united proximal tibia fixed with plate with residual deformity. **B** After removal of the hardware. **C** Removal of the necrotic bone. **D**, **E** Obliteration of the dead space with cement spacer applied on single double leveled K-wire. **F** Postoperative Ap and lateral X-ray

**Fig. 2:**
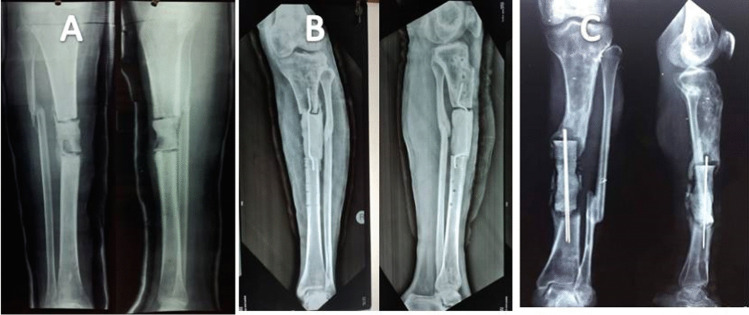
Management of the dead space using PMMA cement spacer. **A** Application of cement spacer without transfixing K-wire **B** Application of the cement spacer on single double level K-wire **C** Application of the cement spacer on double parallel K-wire. Note that in our case series, there is no formal internal or external fixation applied in the first procedure rather than above knee slap. Transfixing K-wire was noticed to provide good stability to enhance the induced membrane formation

Antibiotic-impregnated cement spacer was made by mixing 2 gm vancomycin with 40 gm gentamycin-impregnated cement powder (PALACOS® R + G, Zimmer Biomet). Then formulation of the cement spacer on a double parallel or single double level K-wire was transfixed through the bone gap (Figs. 1 and 2).

A negative pressure wound therapy (NPWT) was applied post-operatively to remove infected transudates. NPWT was applied on the first post-operative day after the first stage until the wound has healed and the soft tissue condition improved.

The PMMA was kept for six to eight weeks with stabilization of the lower limb using a rigid splint. After wound healing, a well-molded walking cast was applied and the patient was allowed partial weight-bearing.

#### Stage 2. Bone and soft tissue reconstruction

In this stage, the cement spacer was removed piecemeal using an osteotome and the K-wires were removed through the bony gap with meticulous preservation of the induced membrane.

A temporary intramedullary K-wire was inserted as a guide for the bone segment during transport in cases with large bone defect. Ilizarov external fixator was applied and a metaphyseal percutaneous osteotomy was performed in the metaphysis furthest from the bone defect. The percutaneous osteotomy was done by multiple drill holes and completed by an osteotome. Then distraction-compression was performed for bone and soft tissue transport to bridge the tissue defect. The rate of distraction-compression was 1 mm per day divided into 0.25 mm every six hours with adjustment of the rate of the distraction-compression according to the follow-up X-rays. The patients started distraction-compression one week after the second stage. The fixator was left in place until complete bone consolidation.

### Evaluation of the results

The results were assessed based on both objective (clinical and radiographic evaluation) and subjective criteria (limb function and patient satisfaction) using our system of results evaluation [15], which is modified from Paley’s evaluation system [16] that combines both radiological and functional outcomes in a strict evaluation system. The outcome is considered satisfactory if the nine evaluation criteria were fulfilled; otherwise, the result is considered unsatisfactory (Table 1). The results were considered satisfactory if the bone and soft tissue were healed without recurrence of infection, < 2.5 cm LLD, < 5° residual deformity, < 5° joint contracture with no or mild pain, and the patient can return to his work. Otherwise, the result was considered unsatisfactory.

**Table 1:** Evaluation of results (modified from Paley et al.)

Parameter	Satisfactory	Unsatisfactory
Bony union	United	Non-united
Residual deformity	Less than 5°	More than 5°
Residual leg-length discrepancy	Less than 2.5 cm	More than 2.5 cm
Recurrent infection	No more infection	Bone and/or soft-tissue infection
Soft-tissue healing	No exposed bone	Soft tissue defect remaining
Permanent joint contracture	Less than 5°	More than 5°
Persistent pain	No or mild pain	Moderate or incapacitating pain
Return to previous work	Yes	Has to change job
Patient satisfaction	Satisfied	Not satisfied

## Results

This study included 32 patients with a mean age of 24 (19–52 years). The mean size of the defect after debridement was 6 cm (range 4–14.5 cm). The average follow-up period was 28 months (range 16–36 months) (Table 2).

**Table 2:** Details of the included cases. Age (years), duration of infection (months), size of bone defect (cm), full weight bearing (months), external fixator index (day/cm), follow-up (months)

Case no	Age	Sex	Etiology	Comorbidities	Duration of infection	Culture	Previous surgery	Site of bone defect	Size of bone defect	Soft tissue defect	Full weight bearing	External fixator index	Follow-up	Results
1	20	M	Open fracture	Smoker	4	MRSA	3	Middle 1/3	4	Yes	4.5	40	16	Satisfactory
2	22	M	Plate fixation	Smoker	12	Klebsiella	2	Upper 1/3	4.5	Yes	4.5	35	20	Satisfactory
3	20	M	Open fracture	Smoker	6	MRSA	2	Middle 1/3	5	Yes	7	45	36	Satisfactory
4	31	M	Plate fixation	Smoker	8	MRSA	3	Upper 1/3	12	Yes	–––	–-	24	Amputation
5	29	M	Open fracture	Smoker	18	Klebsiella	4	Distal 1/3	4	Yes	4.5	40	24	Satisfactory
6	19	M	IM nailing	NA	12	MRSA	2	Distal 1/3	5	Yes	7	45	24	Satisfactory
7	23	M	Plate fixation	Smoker	5	*E. coli*	4	Distal 1/3	7	Yes	7.5	45	24	Satisfactory
8	21	M	IM nailing	Smoker	18	MRSA	3	Middle 1/3	6	Yes	9	48	32	Satisfactory
9	28	F	IM nailing	NA	9	Negative	2	Middle 1/3	8	Yes	10	42	36	Satisfactory
10	26	M	Open fracture	NA	5	MRSA	4	Upper 1/3	4.5	Yes	6	45	28	Satisfactory
11	28	M	Plate fixation	Smoker	4	MRSA	4	Distal 1/3	5	Yes	6.5	45	32	Satisfactory
12	21	M	IM nailing	NA	14	Klebsiella	2	Middle 1/3	6	NA	7	44	36	Satisfactory
13	29	M	Plate fixation	NA	8	MRSA	4	Upper 1/3	6	Yes	6.5	38	18	Unsatisfactory
14	52	M	Plate fixation	HTN	12	Negative	2	Distal 1/3	7.5	Yes	––-	–-	36	Amputation
15	22	M	Plate fixation	Smoker	14	MRSA	4	Distal 1/3	5.5	Yes	6	42	24	Satisfactory
16	23	M	Open fracture	NA	6	MRSA	2	Distal 1/3	5	Yes	8	60	24	Satisfactory
17	21	F	Plate fixation	Smoker	10	*E. coli*	4	Middle 1/3	14.5	Yes	18	40	36	Unsatisfactory
18	24	F	IM nailing	Smoker	24	*E. coli*	4	Distal 1/3	4	NA	4.5	40	36	Satisfactory
19	21	M	Plate fixation	NA	18	MRSA	2	Middle 1/3	7	Yes	9	42	24	Satisfactory
20	25	M	IM nailing	Smoker	28	MRSA	3	Middle 1/3	5	Yes	7	44	24	Satisfactory
21	22	M	IM nailing	NA	24	Klebsiella	2	Distal 1/3	5	Yes	7	48	18	Satisfactory
22	19	M	IM nailing	NA	9	MRSA	2	Distal 1/3	5	Yes	8	52	24	Satisfactory
23	20	M	IM nailing	NA	18	*Staph. a*	4	Middle 1/3	4	NA	5	41	32	Satisfactory
24	28	F	Open fracture	Smoker	7	*E. coli*	4	Upper 1/3	5	Yes	7	44	36	Satisfactory
25	22	M	Plate fixation	NA	4	*E. coli*	2	Upper 1/3	5	Yes	7	45	24	Satisfactory
26	25	M	Open fracture	NA	5	MRSA	3	Middle 1/3	7	Yes	8	45	36	Satisfactory
27	21	M	Plate fixation	NA	10	*E. coli*	4	Middle 1/3	12	Yes	16	45	36	Unsatisfactory
28	19	M	IM nailing	NA	7	Klebsiella	3	Distal 1/3	4	Yes	5	42	36	Satisfactory
29	23	F	IM nailing	Smoker	4	MRSA	2	Middle 1/3	5	Yes	8	50	24	Satisfactory
30	20	M	Open fracture	NA	6	Klebsiella	2	Upper 1/3	4.5	NA	7.5	50	24	Satisfactory
31	19	M	Open fracture	NA	4	MRSA	3	Middle 1/3	4	Yes	7	60	36	Satisfactory
32	25	M	Plate fixation	NA	9	*E. coli*	3	Upper 1/3	6	Yes	8	48	16	Satisfactory

**Fig. 3:**
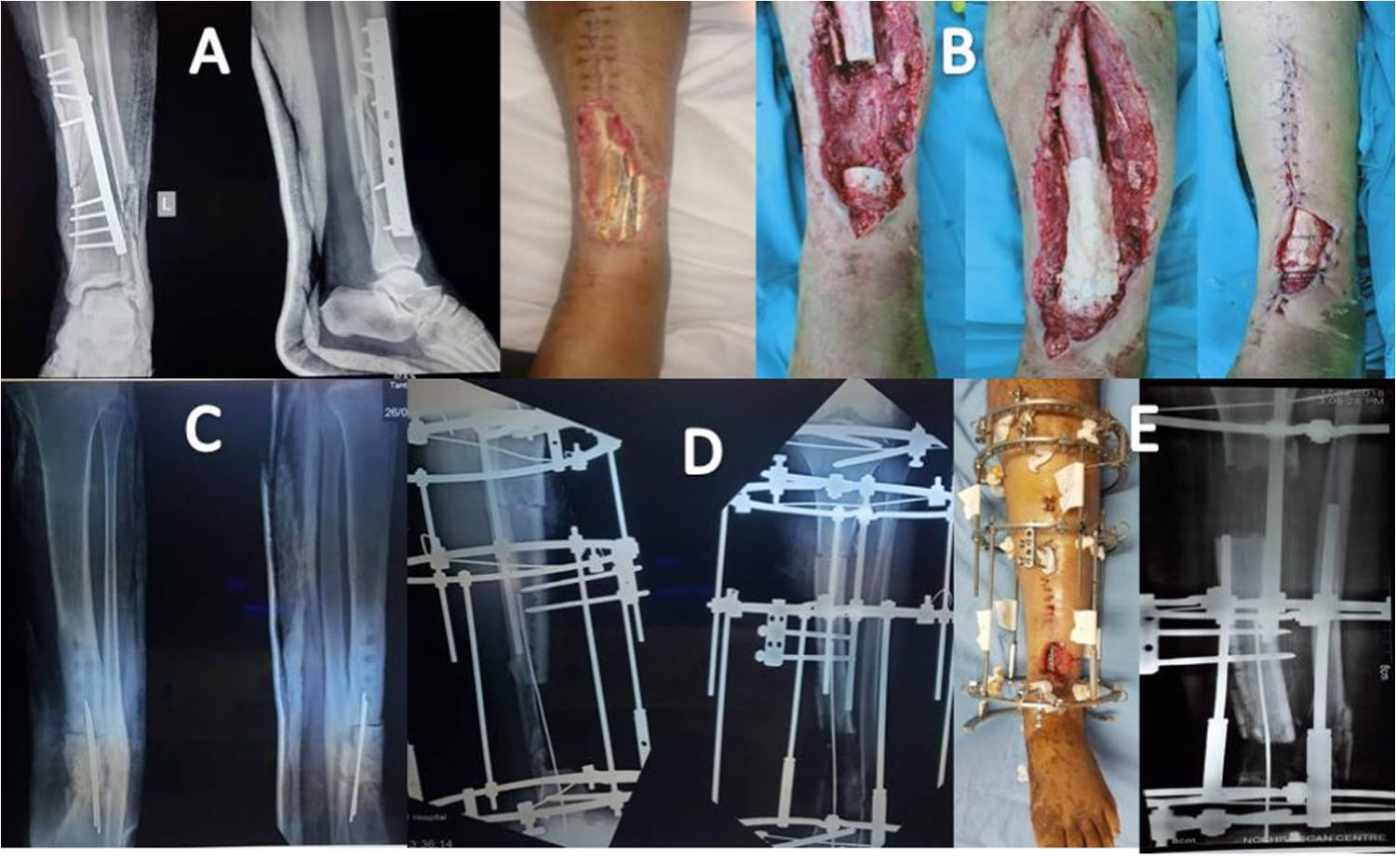
Case of infected nonunion of distal tibia over plate. **A** Ap and lateral X-ray of the Lt leg. Note the soft tissue defect. **B** Phase of debridement and management of the dead space.** C** Application of PMMA on double parallel K-wire. **D**, **E** Bone transport through the induced membrane chamber

**Fig. 4:**
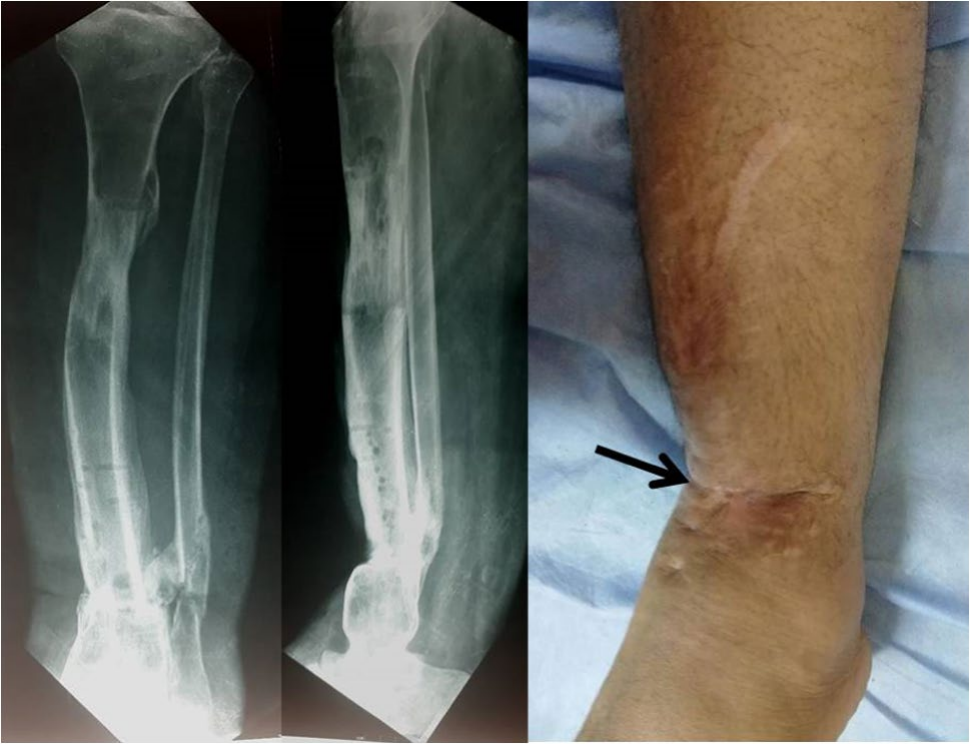
Follow up of the case after removal of Ilizarov external fixator. Note the bone and soft tissue reconstruction (black arrow) was done successfully without the need for further procedures

Successful reconstruction with no recurrence of infection was achieved in 30 cases (94%) without the need for bone or soft tissue grafts (Figs. 3 and 4). Below knee amputation was performed in two cases (6%), one due to intractable infection and the other due to intolerance to the procedure. External fixator index ranged from 35 to 60 days/cm (average 45 days/cm). Superficial pin tract infection was noticed in all cases during the bone transport. It was successfully managed by proper dressing and local pin site care. Oral antibiotics were given when necessary. There were no cases with deep infection or ring sequestra. The functional results were satisfactory in 27/30 cases (90%) and unsatisfactory in 3/30 cases (10%) due to residual leg length discrepancy, joint stiffness, and persistent pain. Two cases presented with ankle stiffness not responding to the physiotherapy with pain during ambulation. One case presented with flexion knee deformity that improved during the rehabilitation. This patient had a leg length discrepancy about 3 cm due to intolerance to the procedure and cessation of limb lengthening. Only one case presented with a refracture at the docking site one year after removal of the frame due to another trauma. It was managed by fixation with an Ilizarov frame and iliac crest bone grafting and the fracture was successfully united.

Results are summarized in Table 3.

**Table 3:** Summary of the results

	*N*	%
Total number of cases	32	100%
Age (years)	19–52y	Average 24 y
Gender	Males: 27 Females: 5	84.4% 15.6%
Previous surgery	32 cases (2–4) operations	100% Average: 3
Etiology	Open fracture: 9 Plate Fixation: 12 IMN fixation: 11	28.1% 37.5% 34.4%
Comorbidities	Smoker: 14 HTN: 1 No comorbidities: 17	43.8% 3.1% 53.1%
Duration of infection (months)	4–28 months	Average: 10.7
Culture	MRSA: 16 *Staph. a*: 1 *E. Coli*: 7 Klebsiella: 6 Negative: 2	50% 3.1% 21.9% 18.8% 6.2%
Site of the bone defect	Upper 1/3:8 Middle 1/3:13 Lower 1/3:11	25% 40.6% 34.4%
Size of bone defect (cm)	4:14.5	Average: 6
Soft tissue defect (Y/N)	Yes: 28 No soft tissue defect: 4	87.5 12.5
Full weigh bearing (months)	4.5:18	Average: 7.5
External fixator index (day/cm)	35:60	Average: 45
Follow-up (months)	16:36	Average: 28
Results	Satisfactory: 27 out of 30 Unsatisfactory: 3 out of 30	90% 10%
Complications	Persistent pain: 2 LLD: 3 Joint stiffness: 2	6.7% 10% 6.7%

## Discussion

Our prospective study demonstrates the effectiveness of combined techniques in the treatment of infected nonunion of the tibia using distraction histogenesis, for bone transport, through an induced membrane chamber in a two-stage procedure. This study was based on the hypothesis that both techniques have their advantages and shortcomings, and combining both techniques may maximize the satisfactory results with avoidance of the undesirable drawbacks of each technique.

Masquelet technique has been accepted for the management of bone defects in infected nonunion with promising results. The PMMA used in the first stage can provide dead space obliteration and high antibiotic elution and allows for membrane formation. The induced membrane contains osteoprogenitor cells and secretes growth factor that helps in revascularization, bone healing, and consolidation [2, 8, 9]. However, it does not deal with the soft tissue defect which is not uncommon in cases of infected nonunion of the tibia. Some of these cases might not be amenable for local soft tissue coverage and may need further complex reconstructive procedures. Masquelet technique also carries donor site morbidity and limited graft availability especially in centres with limited resources due to the non-availability of special equipments for graft harvesting. Complex cases with LLD or limb malalignment cannot be fully addressed using the Masquelet technique which makes the Ilizarov bone transport more suitable in these cases [10].

Distraction histogenesis is used as a procedure of local tissue transport in composite bone and soft tissue defects, avoiding the necessity of complex soft tissue reconstructive procedures [12, 13].

In our case series, successful reconstruction of bone and soft tissue was achieved in 30 out of 32 cases using the Ilizarov bone transport through the induced membrane chamber without the need for further bone or soft tissue grafting.

In his first series of 35 cases, Masquelet reported the need for soft tissue reconstruction with flaps in 28 cases with immediate complications in three flaps which needed further reconstruction using other methods such as Ilizarov procedure [17].

**Fig. 5:**
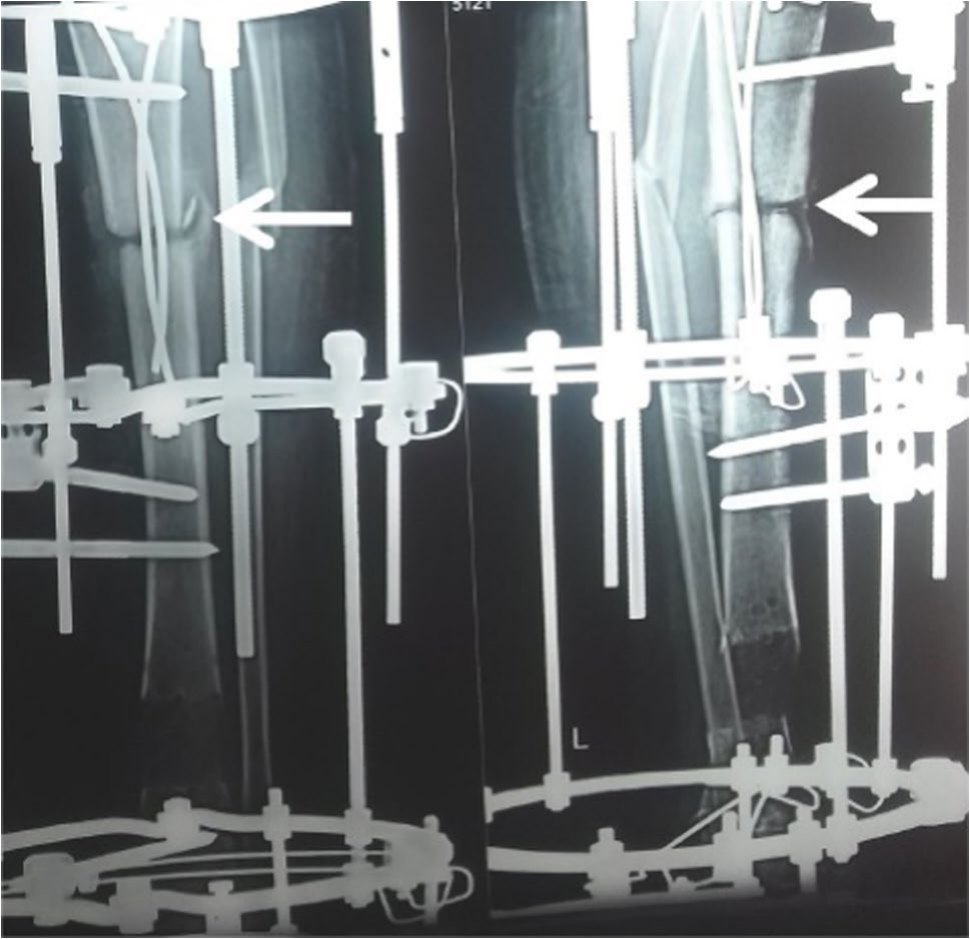
Notice the bridging callus and calcification of the induced membrane at the docking site (white arrows), supporting our assumption of the importance of the induced membrane during bone transport

The main disadvantages of the Ilizarov bone transport are related to the long application of a complex device such as pin tract infections, joint stiffness, regenerate complications, and docking site nonunion [18]. In our series, in the first stage, the authors used the antibiotic cement spacer after radical debridement and square osteotomy without application of the external fixator frame. The patients were allowed for partial weight-bearing in the cast. It was noticed that transfixing the cement spacer with K-wire and augmenting the fixation with only cast provide adequate stability for the membrane formation dispensing the need for fixation with the Ilizarov frame in the first stage and shortening the duration of application of the frame, allowing for psychological rehabilitation of the patients and improvement of the soft tissue condition, hence fewer possibilities of complications related to the long duration of application of the frame. Also, in our hypothesis, the growth factor secreted from the induced membrane and the presence of osteogenic cells can promote rapid bone consolidation in a short duration with fewer complications related to the regenerate and the docking site allowing for early removal of the circular external fixator with fewer morbidities. Moreover, the early formation of a bridging callus and ossification of the induced membrane in some cases during Ilizarov bone transport at the docking site allowed early removal of the Ilizarov frame. This observation supports our hypothesis of the biological advantage of the combination of both techniques (Fig. 5).

Another assumption is that Ilizarov bone transport in a two-stage procedure may carry better results due to improvement of the soft tissue and bone qualities. The argument that a two-stage procedure causes six to eight weeks lost in the first stage can be justified by that such interval allowed for improvement of local soft tissue condition, healing of previous pin sites infection, and resolution of tissue edema. Moreover, the induced membrane chamber hastened docking site healing mostly due to the bioactivity of the membrane. However, this needs further comparative studies with larger case series between Ilizarov bone transport through the induced membrane and Ilizarov bone transport in a single stage and the Masquelet technique in cases of infected nonunion of the tibia.

The main problem in such comparison studies will be the multiple variables in each case such as the site and size of bone defect, either associated with soft tissue defect or not, site and size of the soft tissue defect, bacterial organism, and comorbidities. As every case scenario is unique, this makes the comparison between these groups more complicated.

There are few reports in the literature discussing the combination of Masquelet technique and Ilizarov bone transport [7, 19–21]; however, all of these reports are retrospective in design. Most of these reports discuss the concept of bone transport in two stages from the point of view of combating the infection alone. Unlike other studies, Ilizarov frame in our series applied only in the second stage; this minimizes the duration of the frame application with a lot of benefits for the local soft tissue condition and psychological improvement of the patients.

At the end of the bone transport, there was no need for frame modification or adaptation of bone ends because in the first stage a square osteotomy of bone ends was done. This allowed maximum bone contact at the end of transport. Moreover, the transport was done guided by a temporary intramedullary K-wire in cases of large defect to facilitate the process of transport without deflection of the transported segment. This temporary guide was not fixed to the distal ring due to the fear of the occurrence of impingement during the transport and it was bent at its end and not buried under the skin as it was removed once the transported segment was approaching the docking site at an early stage. Furthermore, the induced membrane—in addition to its biological significance—may represent a guiding chamber during bone transport.

To the best of our knowledge, this is the first prospective study discussing the rationale of combination of both techniques with preservation of the induced membrane as biological chamber for acceleration of bone consolidation, hence early removal of the Ilizarov frame.

In conclusion, in our case series, Masquelet–Ilizarov technique was used for the management of infected nonunion tibia with high satisfactory results without the need for complex soft tissue procedures. This prospective study demonstrates the effectiveness of combined techniques in the treatment of infected nonunion of the tibia using distraction histogenesis, for bone transport, through an induced membrane chamber in a two-stage procedure.
